# Core Self-Evaluations Mediate the Associations of Dispositional Optimism and Life Satisfaction

**DOI:** 10.1371/journal.pone.0097752

**Published:** 2014-06-09

**Authors:** Wensheng Jiang, Fei Li, Haipeng Jiang, Lili Yu, Wenbo Liu, Qiang Li, Luning Zuo

**Affiliations:** 1 Department of Cardiac surgery, Yantaishan Hospital, Yantai, People's Republic of China; 2 Department of Cardiology, Yantaishan Hospital, Yantai, People's Republic of China; 3 Department of Rheumatism, Yantaishan Hospital, Yantai, People's Republic of China; 4 Department of Pediatrics, Yantaishan Hospital, Yantai, People's Republic of China; Cinvestav-Merida, Mexico

## Abstract

**Background:**

Positive traits, such as life satisfaction, optimism, and core self-evaluation (CSE), have garnered increasing attention from researchers and professionals. However, the trilateral relationship among them remains unclear.

**Objective:**

This study examines the effect of dispositional optimism on life satisfaction and primarily verified the mediator role of CSEs.

**Methods:**

Six hundred thirty college students from two general universities completed a questionnaire packet containing life orientation test–revised (LOT–R), core self-evaluations, and satisfaction with life scale. Confirmatory factor analysis (CFA) was conducted to assess the dimension of LOT–R. Bootstrap was used in structural equation modeling to analyze mediation effect.

**Results:**

Results revealed that dispositional optimism and core self-evaluations were significantly correlated with life satisfaction. CFA identified the bidimensional structure of dispositional optimism. SEM indicated that core self-evaluations partially mediated the effect of dispositional optimism on life satisfaction. The final model also revealed significant paths from optimism and pessimism to life satisfaction through core-self evaluations.

**Conclusion:**

The findings extended prior studies and shed light on how dispositional optimism influences life satisfaction. This study provides valuable evidence on how to promote the life satisfaction of human beings in positive psychology. A further study can fully explore the relationship among them in multi-cultural follow-up studies.

## Introduction

Dispositional optimism has received considerable attention in positive psychology. Positive psychology advocates the positive orientation of psychology to motivate the inherent positive strength and excellent quality of people. Scheier et al. considered optimism as a personality trait and called it dispositional optimism [1]. Studies on optimism have focused on one's expectancies for the future [2]. Thus, dispositional optimism refers to the overall expectation of an individual for the occurrence of future positive events, which is a type of attitude toward people and things and significantly affects the cognition and behavior of an individual [3]. Several researchers believe that optimism is a dynamic psychological process that affects the effective analysis of an individual of environment and choices. Studies have shown that young people with optimism have effective choices that can be expressed in terms of cognition, behavior, and attitude. Young people with high dispositional optimism are confident about eventual success by continuous attempt even despite the challenges. By contrast, people who are doubtful try to escape the adversity by wishful thinking. They are drawn into temporary distractions that do not help solve the problem and sometimes even stop trying. Scheier et al. assessed attentional–cognitive strategies as ways of dealing with experiences [4]. More optimists than pessimists make plans for their future and set goals for themselves. Optimists also focus insignificantly on the negative aspects of experiences, such as distress and adversity. Compared with pessimists, optimists gather information about a certain event in the months ahead. Optimists also report the feeling they had benefited from an experience, such as by becoming closer to their spouse.

Optimism is an important internal resource for adjusting psychological and physical health [5]–[8]. Numerous studies have indicated that optimism is closely related to life satisfaction and psychological health. Schweizer and Koch revealed that individual optimism has a significant positive correlation with life satisfaction and a significant negative correlation with depression [9]. The work performance of pressure optimists is better than that of pessimists because optimists and pessimists adopt different strategies for coping with problems; optimists use positive coping strategies, whereas pessimists apply negative coping strategies, such as denial [10]. Lai and Wing investigated laid-off women in Hong Kong and reported that optimism is an important personal resource for dealing with unemployment crisis; women with high optimism separate themselves from the unemployment problem better than those with low optimism and prevent unemployment from damaging their self-concept and self-esteem, thus achieving job satisfaction [11]. He et al. used burn patients as research subjects and proved that optimists have a stronger psychological resilience and they recover from trauma more effectively; thus, they have a higher quality of life compared with pessimists [12]. Contrast cognition and attitude exist between acceptance and active denial. Refusing to accept the reality of a situation, which is common perception and attitude for pessimists, means trying to maintain a worldview that is no longer valid. Optimistic acceptance implies restructuring one's perceptions to come to grips with situations. By accepting that life is compromised (but not over), people develop adaptive parameters within which to live the time left to them. Acceptance may actually serve the purpose of keeping a person goal-engaged and “life-engaged” [2]. Researchers have argued that this attitude can be a start for young people with optimism to improve their abilities and values that help them promote psychological skills, deal with pressure, and adapt to the environment.

A concept highly related to optimism is core self-evaluation (CSE), which is a high-order personality concept that pertains to an individual's benchmark evaluation of his ability and value [13]. CSE involves four personality dimensions, namely, self-esteem, general self-efficiency, neuroticism, and locus of control; these four low-order core traits constitute a potential and broad personality structure [14]–[18]. This concept provides a new perspective in examining the relationship between personality traits and life satisfaction. Researchers have discovered that CSE can directly predict life satisfaction and indirectly affect life satisfaction through the perception of job characteristics, job satisfaction, self-consistency, self-congruence, goal attainment, and other variables. Tsaousis, Nikolaou, Serdaris, and Judge likewise reported that CSE significantly correlates with life satisfaction and adjusts the relationship between physical health and happiness [18]. These studies have uniformly indicated that individuals are satisfied with their lives if they think that they are capable and valuable.

Studies have investigated the relationship between CSE and dispositional optimism, but only few of them have primarily explored this relationship. For instance, Mäkikangas, Kinnunen, and Feldt reported a high correlation between self-esteem and optimism [19]; Kostka and Jachimowicz proved that optimism is significantly correlated with the internal locus of control [20]; Sharpe et al. investigated the relationship between the Big Five personality traits and dispositional optimism and revealed a negative correlation between neuroticism and optimism [21]. The current study evaluated the concurrent effect of CSE and dispositional optimism on life satisfaction by incorporating the relationships among these three variables. To our knowledge, no study has examined the mediator effect of CSE on the relationship between dispositional optimism and life satisfaction. The present study was designed to fill this gap. We hypothesize that CSE mediates the effect of optimism on life satisfaction. To examine the universality of the relationship between optimism and life satisfaction, their relationships in other eastern collective cultures, such as China, must be investigated. Such investigation may show unique characteristics of life satisfaction because of its independent culture and stages of development. In summary, this study aimed to test the mediation effect of CSE between dispositional optimism attributional styles and life satisfaction in collectivism culture and to provide meaningful evidence for external validity of previous findings.

## Method

### 2.1. Participants and Procedure

Participants were 630 undergraduates from two general universities in Jinan and Yantai, China, which consisted of 317 men and 313 women. The ages of students ranged from 19 to 22, with a mean of 20.46 (SD = 1.13). Participants completed the questionnaires in a classroom environment. From the 630 scales that were distributed and collected, 3 unfinished scales were excluded. All the participants were voluntary participated in this study, and provided their written informed consent before completing the measures (guardians on the behalf of the minors signed the informed consent, and provided the overall purpose of this study). After their completed the questionnaire, participants received ¥10 for compensation. The research described in this paper meets the ethical guidelines of Yantaishan Hospital and has been approved by the ethics committee of Yantaishan Hospital.

### 2.2. Instrument

#### 2.2.1. Dispositional optimism (Revised Life Orientation Test (LOT-R)

LOT-R, developed by Scheier, Carver and Bridges, is a 6-item measure (plus 4 filler items) of individual differences in dispositional optimism and pessimism [22]. Items are rated from 1(strongly disagree) to 5(strongly agree). Examples of items include: “In uncertain times, I usually expect the best”, “If something can go wrong for me, it will”.

#### 2.2.2. Core self-evaluation scale (CSES)

The core self-evaluations scale (CSES), which was developed by Judge et al., is a 12-item self-report measure of core self-evaluations [23]. Items are rated from 1 (strongly disagree) to 5 (strongly agree). Examples of items include “I am confident I get the success I deserve in life,” and “Sometimes when I fail, I feel worthless.” The scale scores are the sum of the ratings of the items. Relevant items were reverse-coded.

#### 2.2.3. Satisfaction with Life Scale

The Satisfaction with Life Scale was developed by Diener and Suh, consisting five items on a 7-point rating scale (from 1 =  strongly disagree to 7 =  strongly agree). Example items include: “In most ways my life is close to my ideal” and “I am satisfied with my life”. Scores are the sum of items [24].

### 2.3. Data Analysis

To be sure of the structural relations of the latent structured model, a two-step procedure introduced by Anderson and Gerbing was adapted to analyses the mediation effect [25]. Firstly, the measurement model was tested to assess the extent to which each of the three latent variables was represented by its indicators. If the confirmatory measurement model can be accepted, then the maximum likelihood estimation would be used to test the structural model in AMOS 17.0 program.

The following four indices were used to evaluate the goodness of fit of the model: (a) Chi-square statistic (χ^2^), χ^2^/df, (b) the Standardized Root Mean Square Residual (SRMR), (c) the Root Mean Square Error of Approximation (RMSEA), and (d) the Comparative Fit Index (CFI). In this study, a model was considered to have a good fit if all the path coefficients were significant at the level of 0.05, χ^2^/df was below 5, SRMR was below 0.08, RMSEA was below 0.08, and CFI was 0.95 or more [26].

## Results

### 3.1. Confirmatory Factor Analysis of the Revised Life Orientation Test

**Figure 1:**
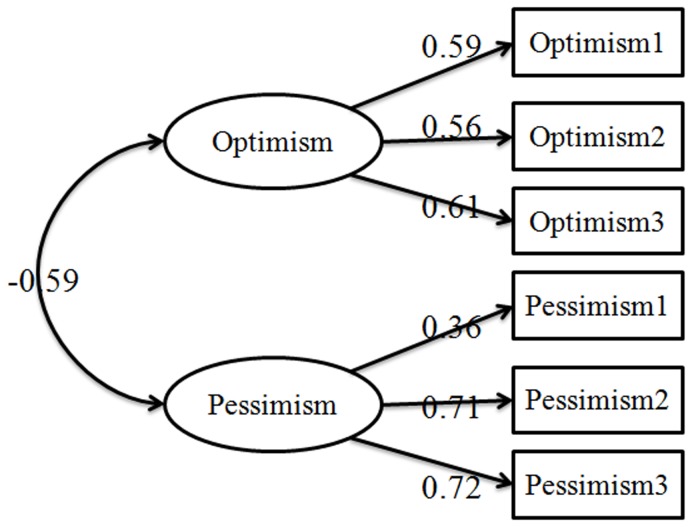
The bidimensional structure of dispositional optimism. Note: Factor loadings are standardized (p<0.05).

The Revised Life Orientation Test was a widely used scale measuring dispositional optimism. However, there were some disputes about the structure of the scale. Scheier regarded dispositional optimism as unidimensional, and optimism and pessimism were the two opposite poles [22]. However, some recent studies have suggested that dispositional optimism is bidimensional and consists of optimism and pessimism factors [27], [28]. So, firstly the confirmatory factor analysis (CFA) was used to assess dispositional optimism was unidimensional or bidimensional. The results revealed that compared with the unidimensional structure, the bidimensional structure fit the data better, see table 1, which suggested that dispositional optimism consisted of optimism and pessimism factors, see figure 1.

**Table 1:** CFA of the Revised Life Orientation Test.

Model	*df*	*χ^2^*	*χ^2^/df*	CFI	RMSEA	SRMR
Unidimensional	9	106.01	11.78	0.837	0.131	0.069
Bidimensional	8	20.45	2.56	0.979	0.050	0.033

### 3.2. Descriptive Statistics and Correlation Analysis

Means, standard deviations, and intercorrelations for all the variables were presented in table 2. The results showed that optimism was positively correlated with core self evaluations (r = 0.290, p<0.01) and life satisfaction (r = 0.476, p<0.01), and was negatively correlated with pessimism (r = −0.402, p<0.01). While pessimism was negatively correlated with core self evaluations (r = −0.359, p<0.01) and life satisfaction (r = −0.415, p<0.01).

**Table 2:** Means, standard deviations, and correlations of the variables of interest.

	Mean	SD	1	2	3	4
1. Optimism	10.75	1.95	1			
2. Pessimism	8.05	1.91	−.402**	1		
3.Core self evaluations	42.42	5.34	.290**	−.359**	1	
4. Life satisfaction	19.68	4.85	.476**	−.415**	.519**	1

Note: *, p<0.05; **, p<0.01.

### 3.3. Measurement Model

CFA was adopted to assess whether the measurement model fit the sample data adequately or not. The fully measurement model included four latent constructs (optimism, pessimism, core self evaluations and life satisfaction) and 12 observed variables. The test of the measurement model came into being a satisfactory fit to the data: χ^2^ (47, N = 627) = 103.82, χ^2^/df = 2.21, p<0.001; RMSEA = 0.044; SRMR = 0.046; and CFI = 0.966. All the factor loadings for the indicators on the latent variables were significant (p<0.001), indicating that all the latent constructs were well represented by their indicators.

### 3.4. Structural Model

**Figure 2:**
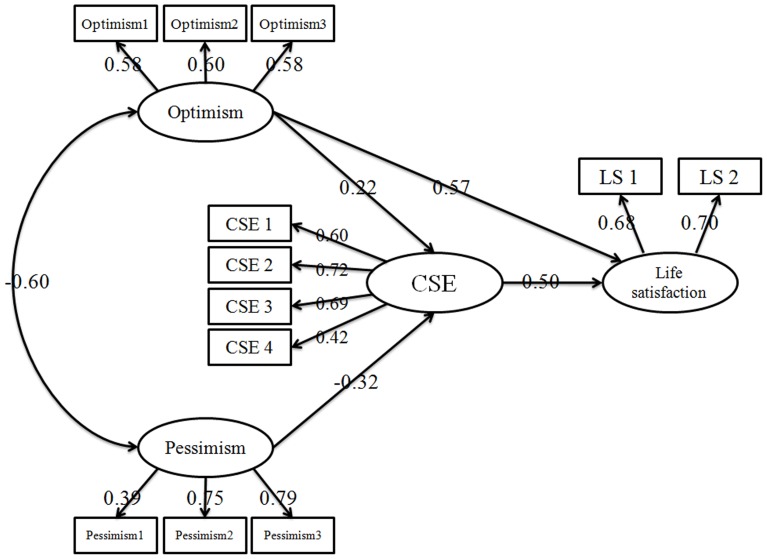
The final structural model (n = 627).

Then SEM was used to analyses the mediation effect. First of all, the direct effect of optimism and pessimism on life satisfaction without the mediator was tested. The directly standardized path from optimism (β = 0.64, p<0.001) and pessimism (β = −0.21, p = 0.008) to life satisfaction were both significantly. Then, a partially-mediated model (model 1) which contained mediator (core self evaluations) and direct paths from optimism and pessimism to life satisfaction was tested. The results showed that the model goodness of fit showed a satisfied fit to the data, χ^2^ (48, N = 627)  = 123.54, χ^2^/df = 2.57, p<0.001; RMSEA = 0.050, SRMR = 0.045, and CFI = 0.954. However, the results revealed that the direct path from pessimism to life satisfaction was not significant. Thus, model 2 was created by delete the insignificant path, as shown in figure 2. The final meditational model showed a good fit to the data, χ^2^ (49, N = 627)  = 124.06, χ^2^/df = 2.53, p<0.001; RMSEA = 0.049, SRMR = 0.044, and CFI = 0.955. The total effect of optimism on life satisfaction through core self-evaluations was 16.18%, while the effect of pessimism to life satisfaction was completely mediated by core self-evaluations.

### 3.5. The Confidence Interval of Direct and Indirect Effects

The mediating effect of core self evaluations between dispositional optimism and life satisfaction was tested for a significance by adopted the Bootstrap estimation procedure in AMOS (a bootstrap sample of 1,500 was specified) [29]. Table 3 shows the indirect effects and their associated 95% confidence intervals. As shown in table 3, optimism had significant direct effect on life satisfaction. In addition, the indirect effects of optimism and pessimism on life satisfaction through core self evaluations were also significant (empirical 95% confidence interval does not overlap with zero).

**Table 3:** Direct and indirect effects and 95% confidence intervals for the final model.

Model pathways	Estimated effect	95% CI Lower bonds	95% CI Up bonds
**Direct effect**			
Optimism→ Core self evaluations	0.221	0.012	0.432
Optimism→ Life satisfaction	0.566	0.408	0.714
Pessimism→ Core self evaluations	−0.319	−0.106	−0.505
Core self evaluations→ Life satisfaction	0.502	0.351	0.677
**Indirect effect**			
Optimism→ Core self evaluations →Life satisfaction	0.111	0.005	0.223
Pessimism→ Core self evaluations →Life satisfaction	−0.160	−0.045	−0.293

## Discussion

This study investigated the concurrent effects of dispositional optimism and CSE on life satisfaction and examined the mediator effect of CSE on the relationship between attributional styles and SWB in Chinese college students. Several findings were obtained in this study. First, the confirmatory factor analysis results provided insight into the structure of life orientation test–revised (LOT–R) in Chinese culture and suggested that this questionnaire is a bidimensional measurement that consists of optimism and pessimism. Previous studies have found that optimistic trait has a significant negative correlation with pessimistic trait. Empirical findings did not support the assumption that optimism and pessimism are polar opposites on a unidimensional continuum. Through exploratory factor analysis, Creed et al. also demonstrated that LOT–R is bidimensional rather than unidimensional and that an insignificant shared variance exists between optimism and pessimism (*r* = 0.16) [30]. In a study by McPherson and Mohr, item extremity manipulations did not change the two-factor structure, and the two-factor model provided a better fit than the unidimensional model [31], [32]. In sum, studies, including the present study, have indicated that LOT–R measures optimism and pessimism simultaneously.

Second, the correlation results showed that optimism correlates with life satisfaction, whereas pessimism has a negative correlation. Evidence from the structural model proved these relationships. This study confirmed that among the Chinese college students evaluated, those with higher scores on optimism were more likely to have higher life satisfaction. Dispositional optimism is a type of continuous and universal positive or negative attitude toward people or things [1]. Naturally, the more people believe that positive events will happen to them and the more they believe that failure only happens in specific situations, the higher life satisfaction they have. Attribution theory indicates that optimists attribute good outcomes to permanent, internal, and global factors and negative events to temporary, external, and specific situations; this behavior leads to their life satisfaction. This finding agrees with those of studies that have adopted different participants to examine the relationship between attributional styles and their effect and have shown that attributional style predicted a state positive effect [33]–[36]. This study provided direct evidence that optimism can help to improve one's life satisfaction. On the contrary, pessimists attribute causes of negative events to global factors and have bleak expectations of future, which proximally cause negative effect on them. Thus, they have a low life satisfaction eventually. Hopelessness theory suggests that people who tend to believe that negative events will happen consistently and that success only happens in random yield failure expectancies that may lead to high negative effect and low positive effect and life satisfaction [17]; thus, pessimists have low life satisfaction.

Third, bidimensional dispositional optimism has different effects on life satisfaction. The path from positive optimism→CSE→life satisfaction was significant and showed that expectation of positive outcomes (optimism) and focusing on the goals set, which may have benefited in enhancing individual's evaluation of his ability and value, eventually enhance one's life satisfaction. The path from pessimism→CSE→life satisfaction was also significant, which might indicate that negative expectations have downside effect on self-evaluation. Likewise, the path from CSE→life satisfaction was significant, which indicated that pessimism has benefits on life satisfaction. Hence, CSE bears part of the mediating effect between optimism and life satisfaction, whereas the effect of pessimism on life satisfaction is totally mediated by CSE. This result indicated that a naively optimistic person might eventually succumb to disappointment in life. As mentioned earlier, CSEs represent a stable personality trait that encompasses an individual's subconscious, fundamental evaluations about themselves, their own abilities, and their own control [17]. Having a significantly high CSE may blind a person to the reality of his abilities that results in poor life outcomes (e.g., “I am confident that I can obtain the success I deserve in life.”). “Negative thoughts” decrease the high CSE that leads to good decision making, life satisfaction, high performance, and positive emotional experience. Positive expectations help to enhance life satisfaction; optimists with high CSE further strengthen their relationship. Pessimists could also have positive attitude toward happiness if they have right self-evaluation. Counseling and interventions in modern life have become important as special interest has developed in the positive aspects of mental health. However, such activities have focused mostly on the positive aspects of optimism and CSE and the negative aspects of pessimism, which may ignore important aspects of each construct. Consequently, these activities become biased. Researchers and counselors should have a dialectical view on these problems.

However, this study has several limitations. First, we regarded optimism as a type of stable personality trait and that its core is an individual's positive expectancy for future events and belief that good results are more likely to occur. Moreover, the theory of optimistic attributional styles coexists with trait optimistic theory [37], [38]. Previous researchers have primarily explored the relationship between optimistic trait and attributional styles [5]; nevertheless, the relationship among attributional styles, trait optimism, personality, and the inner core of subjective happiness should be thoroughly explored. Second, we hypothesized that pessimistic trait has two different paths toward influencing self-evaluation and life satisfaction. However, the results of this study cannot fully confirm this hypothesis. Follow-up research should focus on the positive and negative effects of pessimistic trait. Third, exploring the effects of demographic variables, such as gender, age, and field of study of participants, on optimism, life satisfaction, and CSE will make this study more convincing than its current state. A further study can also explore the effects of demographic variables on the relationships among these variables. Finally, the concepts of optimism and pessimism have distinctive cultural characteristics. Optimism and pessimism are interpreted in western and eastern cultures differently [39]–[41]. The tools used in this study were translated from those of western researchers. Thus, the cultural difference of optimism was neglected to some extent [41]. Therefore, localized tools for evaluating optimism should be developed.
